# Changing Names with Changed Address: Integrated Taxonomy and Species Delimitation in the Holarctic *Colymbetes paykulli* Group (Coleoptera: Dytiscidae)

**DOI:** 10.1371/journal.pone.0143577

**Published:** 2015-11-30

**Authors:** Marcus K. Drotz, Tomas Brodin, Anders N. Nilsson

**Affiliations:** 1 Lake Vänern Museum of Natural and Cultural History, Lidköping, Västra Götaland, Sweden; 2 Department of Ecology and Environmental Science, Umeå University, Umeå, Västerbotten, Sweden; Australian Museum, AUSTRALIA

## Abstract

Species delimitation of geographically isolated forms is a long-standing problem in less studied insect groups. Often taxonomic decisions are based directly on morphologic variation, and lack a discussion regarding sample size and the efficiency of migration barriers or dispersal/migration capacity of the studied species. These problems are here exemplified in a water beetle complex from the Bering Sea region that separates North America from Eurasia. Only a few sampled specimens occur from this particular area and they are mostly found in museum and private collections. Here we utilize the theory of integrated taxonomy to discuss the speciation of the Holarctic *Colymbetes paykulli* water beetle complex, which historically has included up to five species of which today only two are recognized. Three delimitation methods are used; landmark based morphometry of body shape, variation in reticulation patterns of the pronotum exo-skeleton and sequence variation of the partial mitochondrial gene *Cyt b*. Our conclusion is that the Palearctic and Nearctic populations of *C*. *paykulli* are given the status of separate species, based on the fact that all methods showed significant separation between populations. As a consequence the name of the Palearctic species is *C*. *paykulli* Erichson and the Nearctic species should be known as *C*. *longulus* LeConte. There is no clear support for delineation between Palearctic and Nearctic populations of *C*. *dahuricus* based on mtDNA. However, significant difference in size and reticulation patterns from the two regions is shown. The combined conclusion is that the *C*. *dahuricus* complex needs a more thorough investigation to fully disentangle its taxonomic status. Therefore it is here still regarded as a Holarctic species. This study highlights the importance to study several diagnosable characters that has the potential to discriminate evolutionary lineage during speciation.

## Introduction

The application of the biological species concept [1] to geographically isolated forms is a long-standing problem [2–4]. As geographical isolation per se is a reproductive barrier, such forms behave as distinct biological species until they at some point meet due to dispersal or geographical events [5]. This means that the presence or absence of reproductive isolation is controversial and problematic to use as a test for species recognition among strictly allopatric forms [6]. Instead, decisions have to be based directly on the magnitude and the variance of the observed differences. Morphologic variation often provides the only basis for taxonomic hypotheses and decisions, especially in a historical context with the absence of molecular methodologies [7]. Decisions about taxonomic status often pose several problems [8–9], especially when dealing with widely distributed species complexes, where species can show both extensive morphologic and genetic variation [10–14]. Species delimitation will as a consequence of this always include some degree of uncertainty, often ending up being qualified guesses at best [2, 15–16]. This general problem is apparent in the taxonomy of northern terrestrial and freshwater faunas, in which Holarctic distributions of species are often declared [17]. Such decisions are often made disregarding sample size and the effectiveness of barriers separating North America from Eurasia, and the migratory capacity of the actual species being discussed. For many insects and other small animals barriers such as the Bering Strait or the Atlantic Ocean are seemingly very effective isolators [13, 18–21] and intercontinental exchange is more or less dependent on the temporal presence of land bridges [6]. However, on-going gene flow across the Bering Strait has been indicated by the presence of characteristic colour morphs [22] and sequence analyses [23] from adjacent areas of both continents in widespread species. An additional problem that complicates species delimitation of the Holarctic fauna is the lack of genetic and morphologic characterisation of specimens from Siberia and north-east Asia, especially regarding the insect fauna. Therefore taxonomic decisions, within this group, are still strongly dependent on museum and private collections [7] and morphological analyses are predominantly used due to difficulties/restrictions of extracting DNA from such specimens.

Here we apply the ideas of character argumentation and integrative taxonomy on a Holarctic species group of water beetles across the Bering Strait. Water beetles are a very suitable group since they have been thoroughly studied over many centuries, in particularly the morphological variation is well known [24]. During the last 40 years new analytical methods have been introduced that in combination allows for a more thorough decision-base for species delimitations (e.g. population genetic marker [16, 25] and sequence analysis [26]. Consequently, it is well known how these markers vary both within a species complex and between species, both locally and on a wider geographical scale [27]. Here we utilize three well known delimitation methods; landmark based morphometry of body shape, variation in reticulation patterns of the pronotum exo-skeleton and sequence variation of the partial mitochondrial gene *Cyt b*. The mitochondrial gene *Cyt b* is chosen as genetic marker because of its fast evolution, which makes it suitable to study resent speciation events in water beetles [26, 28].

Within the Holarctic, water beetle faunas are relatively speciose with species ranges spanning from circumpolar to isolated occurrences of some species at both sides of the Bering Strait [23, 29]. Especially the aquatic beetle family Dytiscidae stands out due to its high proportion of Holarctic species [23]. Within Dytiscidae there are also examples of forms displaying some intercontinental variation in morphology that is difficult to interpret and species delimitations are then open to subjectivity. Two good examples of such cases are found within the *paykulli*-group of the genus *Colymbetes* Clairville, including relatively large water beetles (body length 16 to 20 mm). This species group is well delimited and was first morphologically recognized by Sharp [24] and later modified by Zimmermann [30]. The number of morphologically delimited species recognized in this group has, however, varied between two and five, and the number of Holarctic species between zero and two (Table 1).

**Table 1:** Taxonomy of the *Colymbetes paykulli* -complex published after all five species names became available.

	Number of species			
Author	Nearctic	Palearctic	Holarctic	Total
Gemminger & Harold 1868	3	2	0	5
Crotch 1873	3	2	0	5
Sharp 1882	3	1	1	5
Leng 1920	3	1	1	5
Zimmermann 1920	2	0	2	4
Larson 1975	2	2	0	4
Zimmerman 1981	0	0	2	2
Larson *et al*. 2000	0	0	2	2

The *paykulli*-group clearly demonstrates how an operational criterion, whichever is chosen, breaks down to an argumentation of its capability of separating evolutionary lineages from each other relative to a temporal and geographic scale [9, 14]. Hence, several perspectives should be integrated and analysed (e.g. phylogeography and comparative morphology) to generate a solid basis for the recognition of separate evolutionary lineages. However, at this point the problem of what characters that should be included in the analyses arises, i.e. identifying characters with the strongest delimitation power [31].

## Material and Methods

### Taxonomic history

Historically, Paykull [32] provided the first published description of a species of the *paykulli*-group. Unfortunately he misidentified the species, later named *Colymbetes paykulli* by Erichson [33], as the *Dytiscus striatus* already described by Linnaeus [34]. Aubé [35] described the 2^nd^ species of the group, named *C*. *dahuricus* based on a single specimen. The type locality was given as ‘Daurie’, an obsolete name for the old mining district near Nertschinsk, today the Eastern part of the Chita Region, between the Schilka and Argun Rivers, in SE Siberia.

Mannerheim [36] was first to recognize the presence of the group in North America, when he described his *Cymatopterus obscuratus* from Kodiak Island, Alaska. Later, LeConte [37] added two more Nearctic species: *Colymbetes longulus* from Lakes Superior and Methy, and *C*. *seminiger* from Saskatchewan.

Both Gemminger & Harold [38] and Sharp [24], in their respective treatments of the Dytiscidae of the world, listed all these five names as valid species, although Sharp [24] placed *obscuratus* among the species that remained unknown to him.

Crotch [39] was seemingly the first to suggest that a species of the group may have a Holarctic distribution, when he commented on page 406 that *C*. *longulus* was “apparently identical with *C*. *paykulli* of Europe, but I have not seen the male.” Sharp [24], however, applied these names to different species, but recorded *C*. *paykulli* from both Europe and western North America. Crotch [39] also erroneously associated *C*. *inaequalis* Horn with the species of the *paykulli*-group. This name is now considered a subspecies of *C*. *densus* LeConte [40].

Zimmermann [41] was first to formally synonymize two names from the group, when in his world catalogue he gave *C*. *obscuratus* as a junior synonym of *C*. *dahuricus*. Later, Zimmerman [40] added two more synonyms (*longulus* = *paykulli* and *seminiger* = *dahuricus*), thus reducing the number of species of the group to only two. All these synonyms were accepted by Larson *et al*. [17], although they stated that the *C*. *dahuricus* synonymy needed re-evaluation.

### Species distinction

**Fig 1:**
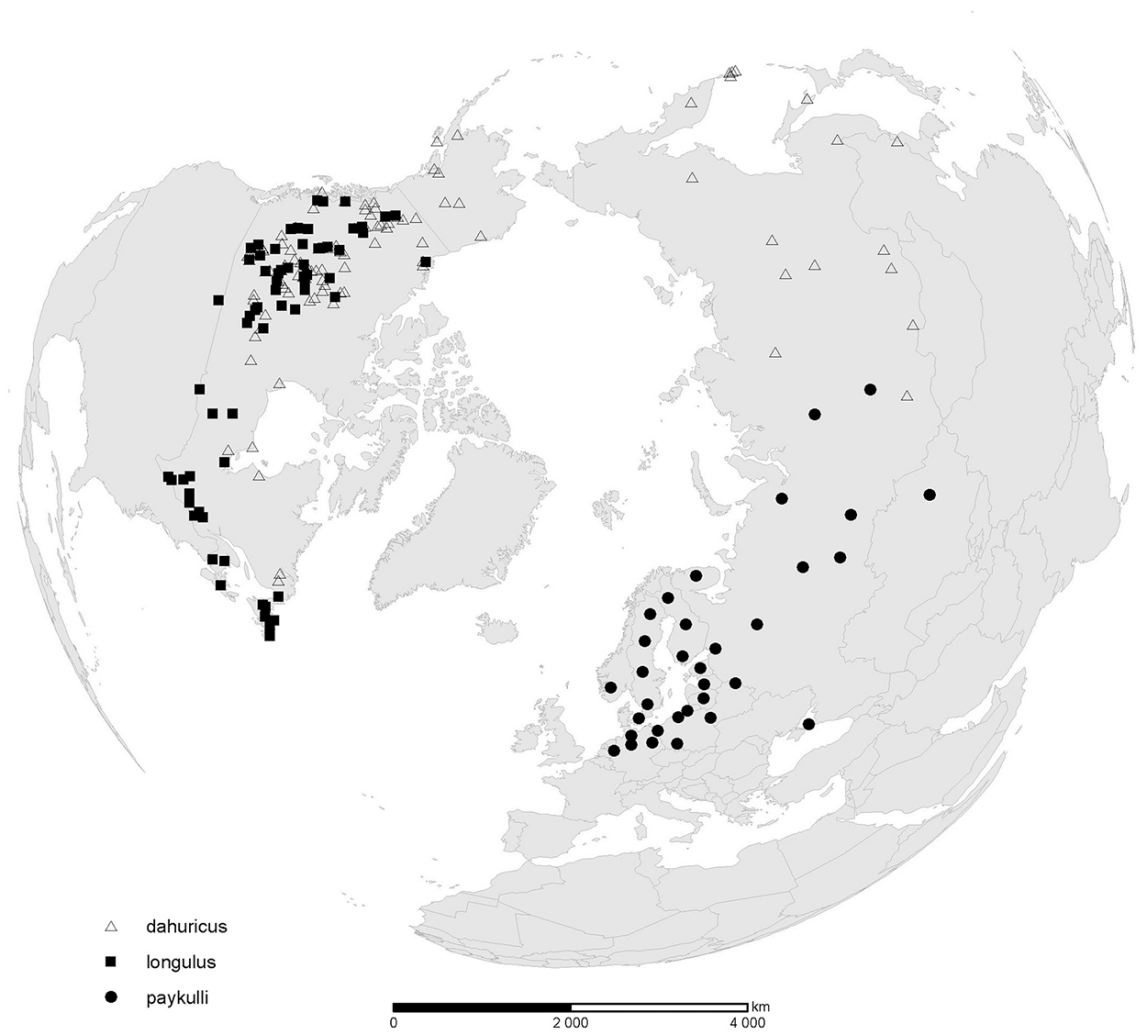
Distribution map of reported observations of the Holarctic (Δ) *Colymbetes dahuricus*, the Palearctic (●) *C*. *paykulli* and the Nearctic (■) *C*. *paykulli*. The latter is within this study accepted as a valid species. This means that the name of the Palearctic species is *C*. *paykulli* Erichson and the Nearctic species should be known as *C*. *longulus* LeConte.

The *dahuricus*-complex: Penis apex rounded and pronotum dark rufous with transverse black discal spot. Geographical variation is seen in penis length, with Palearctic males having longer penis with apex relatively smaller [42] and in the secondary sculpture on pronotum with meshes more transversely stretched in Nearctic specimens [17]. This complex is distributed trans-continentally in North America from Alaska to Labrador with most records from western Canada. It is considered absent from the rest of the USA [17]. In the Palearctic region it occurs mainly east of the Yenisey River with the westernmost records in Altai (Fig 1).

The *paykulli*-complex: Penis apex hooked and pronotum largely black with lateral margins rufous. Geographical variation is seen in the secondary sculpture on pronotum with meshes more transversely stretched in Palearctic specimens [39]. This complex is distributed throughout Canada and from northern US south to Colorado in the Rocky Mts., but not known from Alaska [17]. In the Palearctic region it occurs from the Netherlands and Scandinavia via eastern Europe to Kazakhstan and the Yenisey River in western Siberia (Fig 1).

### Specimens studied

Tissue sample and morphological analyses were based on 351 specimens from 24 regions. The specimens were collected by the authors or borrowed from private or museum collections (Table 2). The Swedish *C*. *paykulli* samples represent local populations (sample B9, J9, K9, L9, T9, S9), whereas all other samples represent a geographical area where specimens from several local sampling sites were pooled (BB, W, ON and BL). In addition the following species were included in the phylogenetic analysis: *Rhantus grapii* (Gyllenhal) used as outgroup, and *Colymbetes exaratus* LeConte, *C*. *fuscus* (Linnaeus), *C*. *schildknechti* Dettner, *C*. *sculptilis* Harris and *C*. *striatus* (Linnaeus) were all included in the ingroup in order to test the monophyly of the *C*. *paykulli*-complex. Collected samples from the Pacific region belong to the Fish Collection, University of Washington in Seattle. Collection samples from Yukon Territory and British Columbia were approved by the St. John's University of Newfoundland, and from Wisconsin by Wisconsin University of Wisconsin-Madison. The samples from Sweden were collected under the permission granted by “The Right of Public Access”, which allows people to pick none-endangered flowers, mushrooms, berries or insects. Specimens collected from Portugal and Alberta, Canada, belongs to the private collections of J. Bergsten, Umeå University and E. Goth-Birkigt, Athabasca University, respectively.

**Table 2:** Species, country, sampling area, number of males and females, sampling code of specimen and collectors are listed. Collectors abbreviations as follows; N. Minakawa (NM), P. Oberg (PO), D.J. Larson (DL), J. Elmberg (JE), A. Töyrä (AT), K.B. Miller (KM), W. Hilsenhoff (WH), M.K. Drotz (MD), E. Goth-Birkigt (EGB), J. Bergsten (JB) and A.N. Nilsson (AN). Samples represent either a geographical area where specimens from several local sampling sites were pooled or a single sample locality. The Nearctic *C*. *paykulli* is within this study accepted as a valid species and should be known as *C*. *longulus* LeConte. See discussion for more information.

Species	Country	Region	♂	♀	Code	Collector
*C*. *dahuricus*	Russia	Sakhalin	8	6	Sk01	NM
	Russia	Kharimkotan	1	2	ON	PO
	Russia	Makanrushi	10	5	ON	NM
	Russia	Onekotan	22	21	ON	PO & NM
	Russia	Shiashkotan	5	3	SA96	NM
	Canada	British Columbia	8	10	BL	DL
	Canada	Yukon Territory	3	5	BL	DL
*C*. *paykulli*	Sweden	Lake Brånsjön	10	10	B9	JE
	Sweden	Lake Jänkkäjärvi	10	10	J9	AT
	Sweden	Lake Kaakkurilammi	10	10	K9	AT
	Sweden	Lake Lompolojärvi	10	10	L9	AT
	Sweden	Lake Takalammi	10	10	T9	AT
	Sweden	Lake Salojärvi	10	10	S9	AT
	Canada	British Columbia	17	11	BB	DL
	Canada	Yukon Territory	2	4	BB	DL
	USA	Colorado	2		W	KM
	USA	Wisconsin	44	29	W	WH
*C*. *dolabratus*	Sweden	Lycksele lappmark	1	2	Cdo	MD
*C*. *exaratus*	Canada	Alberta	1		Cex	EGB
*C*. *fuscus*	Portugal		1	1	Cfu	JB
*C*. *schildknechti*	Portugal		2		Csc	JB
*C*. *sculptilis*	Canada	Alberta	1		Csu	EGB
*C*. *striatus*	Sweden	Ångermanland	2		Cst	AN
*Rhantus grapii*	Sweden	Västerbotten	1	1	RG	AN

### Morphological analyses

We used geometric morphometrics to describe variation in body shape and size differences. Geometric morphometrics utilise the spatial covariation of homologous landmarks to calculate affine (partial warps) and non-affine (uniform) shape components [43]. The shape component is a decomposed description of one specimen in relation to a consensus shape of all specimens in the analysis. Each landmark (geometric position) of a specimen is scaled (centroid size = 1), rotated, translated and aligned to minimise differences between samples. A total of 230 specimens were selected from the total collection to produce equal sample sizes from the different localities. (Table 2). The Procrustes metric (α) was set to null [44], which gives equal weight to partial warps at all spatial scales. The uniform component estimated as described by Rohlf and Bookstein [45] was included in the weight matrix. We used 23 landmarks positioned to capture as much form variation of the beetles as possible: (1) Pronotum, central anterior margin; (2) pronotum, right anteriolateral angle; (3) pronotum, midpoint between landmarks 2 and 4; (4) pronotum, right posteriolateral angle; (5) pronotum, central posterior margin; (6) right elytron, right anteriolateral angle; (7–11) abdomen, right lateral intersegmental fold between metacoxal plate and abdominal segments 1 to 5; (12) right elytron, posterior apex; (13) left elytron, posterior apex; (14–17) abdomen, left lateral intersegmental fold between abdominal segments 5 to 1; (18) abdomen, left lateral intersegmental fold between metacoxal plate and abdominal segments 1; (19) left elytron, right anteriolateral angle; (20) pronotum, left posteriolateral angle; (21) pronotum, left midpoint between landmarks 20 and 22; (22) pronotum, left anteriolateral angle; (23) scutellum, posterior apex. Landmarks were not used on the head as its position relative to the rest of the body may obscure the analysis [46–48]. A Summa Sketch III (Summagraphics) graphics tablet was used to capture the geometric position of each landmark relative to a Cartesian coordinate system. To assess the repeatability and minimise measurement errors of the geometric morphometric analyses, each specimen was measured three times [16, 49]. Measurements were made by the same person.

Morphological variation within species and sexes was analysed with a Principal Component Analysis (PCA) of the partial warps, the uniform components and centroid size of the body in the Relative warps program v 1.20 [50]. The obtained components (*relative warps*) were only used to describe shape variation [51–52] and to calculate repeatability as described by Arnqvist and Mårtensson [53].

### Microsculpture analyses

The pronotal microsculpture was classified into four groups based on the following criteria; (1) Secondary microsculpture represented by narrow stretched meshes strongly transverse in arrangement along the entire central line of the pronotum. Sculpture does not include any small, round and closed cells. (2) Secondary microsculpture represented by narrow stretched meshes strongly transverse in arrangement present only in front of the central line. The secondary sculpture posterior along the central line forms rounded closed cells, which do not show a distinct transverse pattern. (3) Secondary microsculpture represented by small closed cells on both sides of the entire central line. In front of the central line a more stretched pattern may be observed. (4) Secondary microsculpture represented by numerous very small, rounded and closed cells. No observation of a more directed pattern was made.

Differences in the distribution of classes between sexes across the two candidate species was tested using chi-square tests of homogeneity. The *Yates' correction for continuity* is employed to improve the accuracy of the null-condition sampling distribution of chi-square [54].

### DNA amplification and sequencing

DNA was extracted from one hind leg or the thoracic flight muscles of the frozen or alcohol preserved beetles, using a Qiagen DNeasy protocol for animal tissues. Partial cytochrome b (*Cyt b*) sequences were amplified with the two flanking primers CB-J-10933 and CB-N-11367 as described in Simon *et al*. [55] using the following polymerase chain reaction (PCR) program: denaturation 94°C (90 s), annealing at 50°C (30 s) and extension at 72°C (60 s). This cycle was repeated 30 times, followed by an extension period of 5 minutes. The PCR products obtained were single clear bands with no signs of non-specific amplification. The amplified product was approximately 430 bp in length. The product was run on a 1.0% agarose gel and then removed from the gel and purified with a Jetsorb DNA extraction kit. Sequencing reactions were performed with the DYEnamic ET terminator kit. Each sequence was sequenced from both its 3' and 5' ends. Corresponding GenBank accession numbers for the partial mtDNA sequences are KT368698-KT368722.

### Phylogenetic analysis

The mtDNA sequences were aligned with the ClustalW multiple alignment option in BioEdit, version 4.8.10 [56]. No gaps were inserted within the alignment. The evolutionary history of the Neartic and Paleartic *Colymbetes paykulli* and *C*. *dahuricus* was inferred by using the Maximum Likelihood method. The phylogenetic analysis is based on the partial *cytochrome b* gene, rooted with *Rhantus grapii*. Initial trees for the heuristic search were obtained by applying Neighbor-Join and BioNJ algorithms to a matrix of pairwise distances estimated using the Maximum Composite Likelihood (MCL) approach, and then selecting the topology with superior log likelihood value. Maximum Likelihood method was based on the Hasegawa-Kishino-Yano model (*HKY*) [57] with an optimized discrete Gamma distribution (*G*) with a rate differences among sites in 5 categories and invariable sites (*I*). Bootstrap values were calculated with 300 replicates. The Maximum Likelihood analysis involved 25 nucleotide sequences of a length of 353 positions. Analyses were conducted in MEGA6 [58]. Nodal support within the phylogenetic trees was assessed with bootstrap percentage after 1000 re-sampling steps [59]. To evaluate if the data set was subjected to ‘long branch attraction’ we compared the strict consensus tree topology between two phylogenetic analyses; the first including all sequences including the outgroups and the second analysis including only the sequences from the ingroup as described by Bergsten [60]. Unweighted parsimony analysis was performed by applying heuristic search with tree bisection-reconnection branch swapping. A total of 3000 searches were done with 100 replicates and ten random-addition sequence iterations per search started from a random tree. All characters were non-additive, and uninformative characters were excluded before the analysis. The above analyses were run in WinClada ver. 0.9.99 [61]. The molecular clock test was performed by comparing the ML value for the tested topology with and without the molecular clock constraints under Kimura 2-parameter model [62]. Evolutionary analyses were conducted in MEGA5 [63]. Putative species boundaries were tested with the Poisson tree processes (PTP) model on the phylogenetic input tree from the Maximum Likelihood sequence analysis. The PTP model utilize the phylogenetic species concept [64] with the fundamental assumption that the number of substitutions between species is significantly higher than the number of substitutions within species [65].

## Results

### Morphological analyses

**Fig 2:**
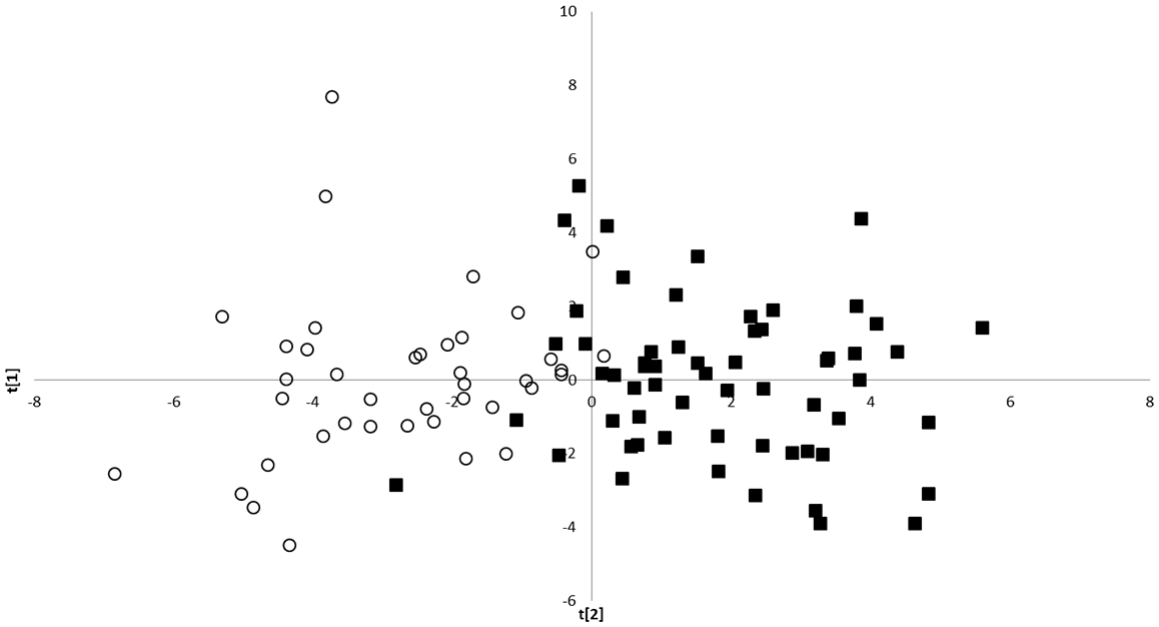
Morphological shape variation between and within *Colymbetes paykulli* males from the Palearctic (○) and Nearctic (■) regions. Shape variation was analysed with a Principle Component Analysis (PCA) of the partial warps, the uniform components and centroid size of the body in the Relative warps program v 1.20 (Rohlf 1998). The shape difference between the regions is significant (first component d.f. = 86, t-value = 13.30 p<0.001). The Nearctic *C*. *paykulli* is within this study accepted as a valid species and should be known as *C*. *longulus* LeConte.

The repeatability of the first five relative warps and centroid size ranges from 85.0 to 99.7% in the *C*. *paykulli* males and from 80.0 to 98.4% in females. This clearly indicates that the variation in form found here is due to true form variation and not measurement errors. The Nearctic *C*. *paykulli* males were more compact and had a wider abdominal apex than the Palearctic males, whereas the form variation was opposite in the females. We found a small morphological overlap in both sexes of this species-complex (Fig 2), but the difference in morph space was significant in both sexes (t-test of the first component: males (d.f. = 86, t-value = 13.30) *P*-value<0.001, and females (d.f. = 74, t-value = -5.67) *P*-value< 0.001). A larger morphological overlap between populations was seen in *C*. *dahuricus* males than in males of *C*. *paykulli* (T-test of first component (d.f. = 18, t-value = -5.01) p<0.001). As a result of too few *C*. *dahuricus* females to generate representative data they were subsequently excluded from the analysis.

### Reticulation diversity

In the Palearctic *C*. *paykulli* males and females secondary reticulation classes of the pronotum was not significantly differentiated (Yates' chi-square: 0.011, Yates' *P*-value: 0.995), but the reticulation class 2 was more frequently observed than class 1,3 and 4 (Table 3). Significant difference was observed between the Palaearctic and Nearctic samples in both males and females reticulation patterns, since only reticulation class 4 was found on specimens from the Nearctic region (males: d.f. = 3, Yates' chi-square: 72.88, Yates' *P*-value: <0,001; Females: d.f. = 3, *P*-value: <0.001, Yates' chi-square: 72.65). Significant differences between the Palaearctic and Nearctic *C*. *dahuricus* in reticulation patterns were observed in both sexes (Males: d.f. = 1, Yates' chi-square: 7.912, Yates' *P*-value: 0.005; Females: d.f. = 1, Yates' chi-square:4.267, Yates' *P*-value:0.039). However, in the Nearctic *C*. *dahuricus* both reticulation class 2 and 3 were found in both sexes, whereas only class 2 was observed on the Palaearctic specimens.

**Table 3:** The pronotal microsculpture within both sexes in the C. *paykulli*-complex. Microsculpture was classified into four groups which are described in the material and method section Microsculpture analyses. The Nearctic *C*. *paykulli* is within this study accepted as a valid species and should be known as *C*. *longulus* LeConte. See discussion for more information.

Area	Spec	Sample	Females				Males			
			1	2	3	4	1	2	3	4
Palearctic	*C*. *paykulli*	B9	2	7	1	0	1	8	1	0
	*C*. *paykulli*	J9	3	7	0	0	0	10	0	0
	*C*. *paykulli*	K9	4	6	0	0	4	6	0	0
	*C*. *paykulli*	L9	1	9	0	0	1	8	1	0
	*C*. *paykulli*	T9	1	7	2	0	3	7	0	0
	*C*. *paykulli*	S9	1	9	0	0	2	8	0	0
Nearctic	*C*. *paykulli*	BB	0	0	0	10	0	0	0	10
	*C*. *paykulli*	W	0	0	0	10	0	0	0	10
Palearctic	*C*. *dahuricus*	ON	0	10	0	0	0	10	0	0
Nearctic	*C*. *dahuricus*	BL	0	5	5	0	0	3	7	0

### Sequence variation

The molecular clock test with the null hypothesis of equal evolutionary rate throughout the tree was not rejected at a 5% significance level (*P*-value < 0.176). The mtDNA *Cyt b* data set included 353 base-pairs. A total of 95 sites were parsimony-informative over all taxa, 91 within the genus *Colymbetes*, and 41 within the *C*. *paykulli*-group. Translation of nucleic acid to amino acid sequences revealed that most of the variation seen represent silent mutations.

The uncorrected p-distance between haplotypes of Nearctic *C*. *paykulli* was 0.011 and in Palearctic *C*. *paykulli* 0.003–0.008, whereas the difference between the two regions was almost ten times higher (0.074–0.079). A comparatively smaller difference was seen in both the Nearctic *C*. *dahuricus* 0.003 and between haplotypes from the Palearctic region 0.003–0.008. The uncorrected p-distance between haplotypes of *C*. *dahuricus* from the Nearctic and Palearctic regions (0.040–0.048) was only about half of that in *C*. *paykulli*. The difference between haplotypes of the western Palearctic *C*. *schildknechti* and *C*. *fuscus* was 0.110–0.144.

### Phylogenetic analysis

**Fig 3:**
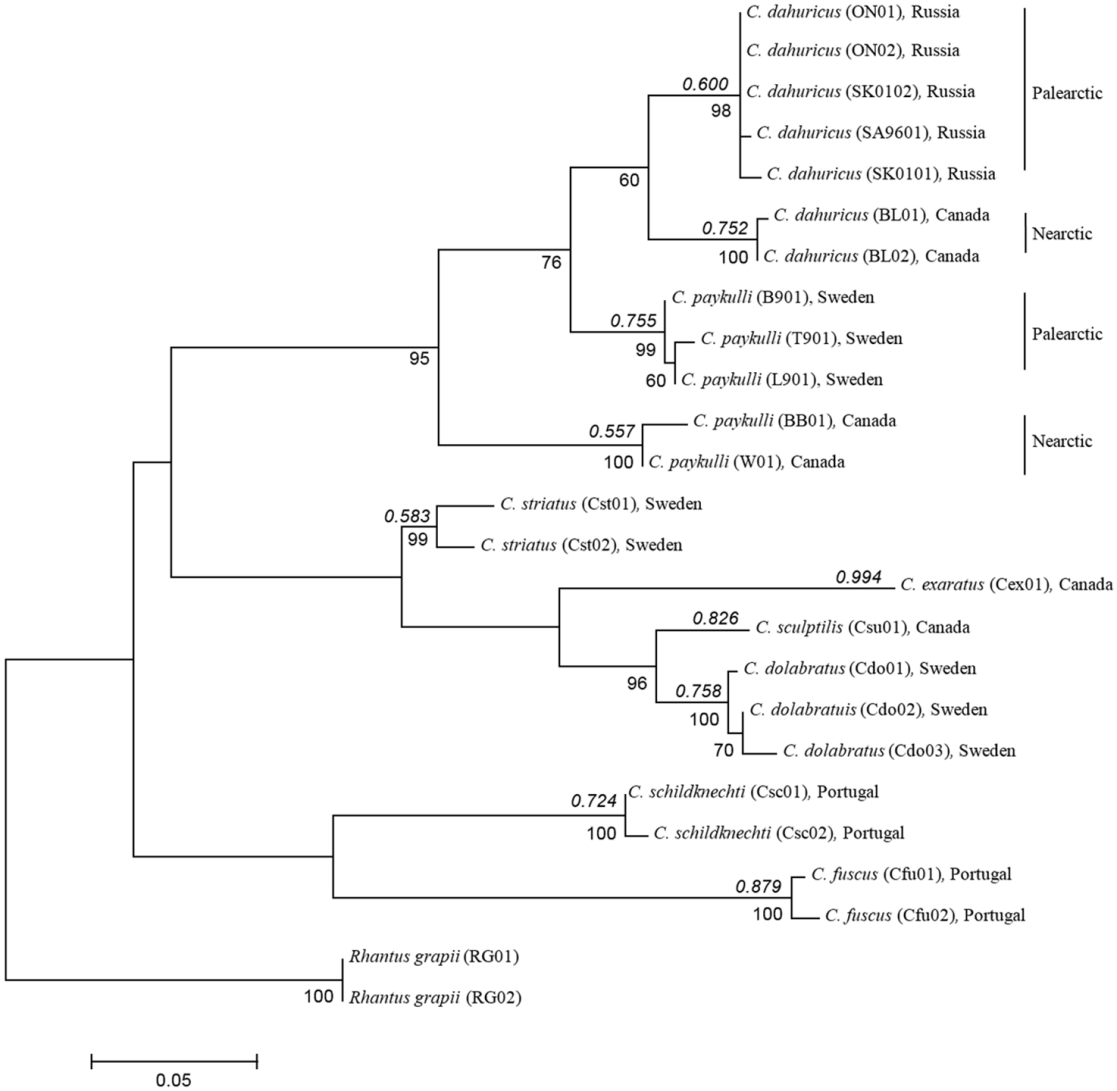
The evolutionary history of the *Colymbetes paykulli* complex was inferred by using the Maximum Likelihood method based on the Hasegawa-Kishino-Yano model with a discrete Gamma distribution (5 categories (+*G*, parameter = 0.3301)) and a invariable rate variation model ([+*I*], 58.5832% sites). The tree with the highest log likelihood (-1493.9031) is shown. Bootstrap values above 60% are reported below branches. Above branches are the Maximum likelihood partition support values from the Poisson tree processes (PTP) model given for each species identified by the model. Sequence reference to individual sample from population follow table 2. The Nearctic *C*. *paykulli* is within this study accepted as a valid species and should be known as *C*. *longulus* LeConte.

The Maximum Likelihood method based on the HKY+*G*+I model resulted in a tree with the highest log likelihood -1493.9031. Bootstrap values above 60% are reported below braches (Fig 3). Gamma distribution (+*G*) was estimated to 0.3301. The invariable rate value (+*I*) was estimated to be 58.5832% per site. Parsimony analysis did not support that the data was subjected to ‘long branch attraction’. The analysis resulted in four most parsimonious (MP) trees with a length of 204 steps (uninformative characters excluded), and CI and RI of 0.54 and 0.79, respectively (tree not shown). The subsequent phylogenetic analysis without the out-group species *Rhantus grapii* resulted in two MP un-rooted trees with a tree length of 181 steps, CI and RI of 0.59 and 0.81, respectively (tree not shown). The main differences between these six fundamental trees from the two analyses were the position of the clade including *Colymbetes fuscus* and *C*. *schildknechti* and the clade including the *C*. *exaratus* haplotype and the Swedish *C*. *striatus*. The topology of the clade including all the *paykulli*-group specimens was identical in the two analyses. When the outgroup species was excluded, the resulting topologies were identical to two of the four most parsimonious trees in the complete analysis.

### Phylogeography

A clear geographical subdivision between *Colymbetes paykulli* and *C*. *dahuricus* was observed within the Maximum Likelihood tree (Fig 3). The basal positioned Nearctic *C*. *paykulli* haplotypes constitutes the sister-group to a group (BS value of 76%) that was subdivided into one clade containing haplotypes only from the Palearctic *C*. *paykulli* (BS value of 99%) and another group containing all *C*. *dahuricus* haplotypes (BS value of 60%). The former clade consisting of *C*. *dahuricus* haplotypes was geographically and haplotypically subdivided into a Nearctic (BS value of 100%) and a Palearctic group (BS value of 98%). These results are congruent with the geographical distribution of the species detailed in the introduction. The Nearctic *C*. *paykulli* is reported across the entire North American continent except for Alaska, whereas all known records of the Palearctic *C*. *paykulli* occur west of the Yenisey River and hence not reaching Beringia.

The Poisson tree processes (PTP) model identified ten putative species boundaries within the total sample (Table 2), excluding *Rhantus grapii*, based on the number of substitutions occurring between and within species. Maximum likelihood partition support values are given for each species on the maximum likelihood tree shown in Fig 3. The model suggests that *C*. *paykulli*, just as *C*. *dahuricus*, consists of two Nearctic and Palearctic putative species. The overall result of this study is that the Nearctic *C*. *paykulli* should be and is within this study accepted as a valid species that has to be known as *C*. *longulus* LeConte. See discussion for more information.

## Discussion

Intuitively, based on the large geographical distance and low dispersal probability between the Nearctic and Palearctic region, it seems likely that there should be three or four species within the *Colymbetes paykulli* Holarctic complex instead of the now recognized two widespread species. The occurrence of *C*. *dahuricus* on both side of the Bering Sea and on small volcanic islands within this region suggests that dispersal, and thereby some degree of gene flows, is possible between the Nearctic and Palearctic region. Contrastingly, the vast distance between populations of *C*. *paykulli* in the two regions makes inter-population dispersal unlikely and hence very likely nullifies the likelihood for gene flow between the populations. This however, has yet to be quantitatively supported. Small sample sizes, as pointed out in the introduction, often reduce the accuracy of decisions regarding species delimitation. Especially, as scientists usually do not know a priori when evolutionary lineage separated. Such knowledge is likely to influence expectations regarding the differentiation between evolutionary lineages in various traits [66]. Here we will discuss our results in relation to different species delimitation methods and their taxonomic implications [14].

### Geographic distance and sequence variation

We show that the geographical distribution of *C*. *paykulli* on the two continents (Fig 1) is congruent to the result of the phylogenetic analyses of the cytochrome b (*Cyt b*) sequences and PTP model (Fig 3). The uncorrected p-distance, given in the Results section, between the haplotypes that contain the Nearctic *C*. *paykulli* and the clade containing the Palearctic *C*. *paykulli* and all *C*. *dahuricus* indicates a divergence time around 3.9 MYA given that a sequence divergence of 2%, which is regarded as normal within in insects [67], equals a separation time of 1 MYA. The ancestors that gave rise to the Nearctic and Palearctic *C*. *dahuricus* probably separated around 2.4 MYA. These dates lie well within the time–range following the first opening of the Bering Strait land connection 5.4 MYA [68–69]. The land bridge is, however, tightly linked to glaciation periods and sea level variation which means that the land-bridge repeatedly reoccurred during the last 4.5 MYA. The most recent land bridge existed as a vast tundra plain until about 9000 b.p., when the rising sea level flooded the area [70]. In addition the vast tundra plain, during the Bering Strait land connection, was most likely a suitable habitat for water beetles with a large number of standing water bodies [71] that could act as stepping stones during dispersal [72]. The dispersal of *Colymbetes* is poorly studied, but similar sized and smaller water beetles are known to disperse several hundred meters or even further [73–75], and the fact that *C*. *dahuricus* exists on some of the fairly isolated Kuril islands clearly shows its dispersal potential. Based on this it is assumed that multi-dispersal opportunities across the former land bridge was possible. However, given the relative low number of specimens in our analysis clear species delimitation is difficult. But there is an indication, based on the *cyt b* gene, that the haplotype lineages within *C*. *dahuricus* separate the Palearctic and Nearctic regions. A similar argument applies to *C*. *paykulli*. The *Cyt b* gene is especially interesting since it, in contrast to other more commonly used mtDNA genes as the cytochrome c oxidase subunit 1 (*COI*), has proven useful in separating beetle species of more recent origin [26– 27].

### Reticulation variation

The pronotum secondary reticulation displays a clear variation in the observed patterns within the Palearctic *C*. *paykulli* population samples B9, J9, K9, L9, T9 and S9 (Table 3). This variation is absent in the Nearctic *C*. *paykulli* samples, although the specimens representing sample BB and W are taken from several different populations across a wider geographical range. This strongly supports a delimitation of these two evolutionary lineages. A similar degree of variation is observed within the diving beetle *Agabus bipustulatus* across the west Palearctic on the secondary reticulation on the elytra. In this study the reticulation variation was genetically connected with the evolution of different mountain ecotypes [27]. The observed reticulation variation in *C*. *dahuricus* across the Nearctic and Palearctic region is overlapping and hence does not support differentiation, even if the differentiation is significant (Table 3).

### Size and shape differences

The landmark based morphometrics method [43] has earlier been used to capture geographical form variation in water beetles across large areas in relation to species delimitation [16, 48, 76]. A specific strength of this method is the possibility to visualize shape variation [77]. Here we use specimens of *C*. *paykulli* and *C*. *dahuricus* from different regions in order to maximize the morphological variation. Despite this we observe a significant differentiation between C. *paykulli* and *C*. *dahuricus* across the Nearctic and Palearctic, a result that supports a delimitation of *C*. *paykulli* and *C*. *dahuricus* into four evolutionary lineages (Fig 3).

## Conclusion

The conclusion of this work is that the Palearctic and Nearctic populations of *Colymbetes paykulli* sensu Zimmerman [40] are rightly given the status of separate species. This conclusion is based on the fact that all tree delimitation methods: morphologic, genetic and reticulation show significant separation of between the Palearctic and Nearctic populations. This means that the name of the Palearctic species is *C*. *paykulli* Erichson, whereas the Nearctic species should be known as *C*. *longulus* LeConte. For *C*. *dahuricus* we do not find such clear cut separation between Palearctic and Nearctic populations. Here two methods, genetic and reticulation pattern, show no clear support for a delimitation of *C*. *dahuricus* into two species. There is, however, a fairly large difference in sample-size between the two regions. The combined conclusion is that more studies of the *C*. *dahuricus* complex are needed to fully comprehend its taxonomic status. Therefore it is here still regarded as a Holarctic species. Our findings highlight the importance, supporting the conclusions from de Queiroz [9] and Dayrat [31], of studying several potentially important diagnosable characters in combination to discriminate evolutionary lineage at different time scales during speciation.
